# CircCYP24A1 hampered malignant phenotype of renal cancer carcinoma through modulating CMTM-4 expression via sponging miR-421

**DOI:** 10.1038/s41419-022-04623-0

**Published:** 2022-02-26

**Authors:** Xiaorong Wu, Jiale Zhou, Ling Zhao, Zhaolin Yang, Chen Yang, Yonghui Chen, Wei Xue

**Affiliations:** 1 Department of Urology, Renji Hospital, School of Medicine, Shanghai Jiaotong University, Shanghai, China; 2 Department of Pathology, Renji Hospital, School of Medicine, Shanghai Jiaotong University, Shanghai, China; 3 Department of Urology, Huashan Hospital, Fudan University, Shanghai, China; 4 Fudan Institute of Urology, Huashan Hospital, Fudan University, Shanghai, China

**Keywords:** Renal cell carcinoma, Cancer metabolism

## Abstract

Renal cell carcinoma (RCC) is a lethal urinary malignancy. Circular RNAs (circRNAs) contribute to the malignant phenotype and progression of several types of human cancers, including RCC. In this study, we identified relatively low hsa_circ_0060927 (circCYP24A1) expression in RCC tissue through high-throughput sequencing and RT–qPCR. Fluorescence in situ hybridization (FISH) was used to validate the expression and subcellular localization of circCYP24A1 in RCC tissues. CCK-8, Transwell, EdU, and wound-healing assays indicated that circCYP24A1 overexpression inhibited the proliferation, invasion, and migration of RCC cells. Dual-luciferase reporter, RNA immunoprecipitation (RIP), FISH, and RNA-pulldown assays verified that circCYP24A1 inhibited RCC progression by sponging miR-421, thus inducing CMTM-4 expression. Xenograft assays and metastasis models further indicated that circCYP24A1 significantly inhibited the metastasis and proliferation of RCC cells in vivo. Taken together, circCYP24A1 is a prognosis-related circRNA in RCC that functions through the circCYP24A1/miR-421/CMTM-4 axis to modulate RCC progression.

## Introduction

Renal cell carcinoma (RCC) is the 7th most common tumor worldwide [1] and is related to approximately 140,000 deaths per year. Surgery remains the main treatment for RCC [2]. However, recurrence still occurs in 30% of patients with RCC after radical nephrectomy. Moreover, resistance to radiation therapy and conventional chemotherapy leads to an unsatisfactory clinical outcome of RCC [3]. The high recurrence rate and unclear pathogenesis still intrigue us and prompt explorations to improve the therapeutic and diagnostic strategies for RCC.

CircRNA was first explored using electron microscopy 50 years ago; circRNA is generated by backsplicing to form a circular configuration and is more stable than linear RNA [4]. The competitive endogenous RNA hypothesis is a conventional and well-recognized mechanism by which circRNAs regulate biological processes. Acting as ceRNAs, circRNAs interact with their miRNA targets and function as miRNA sponges, modulating expression at the posttranscriptional level [5].

Circular RNAs (circRNAs) were shown to be closely correlated with the progression of RCC in previous studies [6, 7]. A study reported that circRNA cRAPGEF5 suppresses RCC via the miR-27a/TXNIP pathway [8]. Additionally, circTLK1 sponges miR-136-5p to promote RCC progression [9]. As circRNAs function diversely as tumor promoters or suppressors in RCC, an urgent need is to characterize and investigate additional as-yet undiscovered circRNAs.

Here, we first found that circCYP24A1 plays a crucial role in RCC. High circCYP24A1 expression was associated with favorable clinical outcomes of RCC. As circRNA mainly regulated downstream miRNA through ceRNA-regulation mechanism, we further identified miR-421 as the target of circCYP24A1.MiR-421 is proven to be a tumor promoter through cell-proliferation enhancement and cell-apoptosis inhibition in several malignancies and correlates with the prognosis of patients with osteosarcoma and lung cancer [10, 11]. CKLF-like MARVEL transmembrane-domain-containing family(CMTM) serves as a novel tumor suppressor in malignant progression. As the most conserved member of the CMTM family, CMTM4 is frequently downregulated and regulates cell growth and cell cycle to exert tumor-suppressing activity in several cancers, including RCC, by inhibiting the AKT and ERK pathways [12, 13]. Furthermore, circCYP24A1 suppresses RCC progression by acting as a ceRNA to sponge miR-421 and increase the expression of CMTM4, which resulted in improved antitumor function. We suggest that circCYP24A1 may represent a potential therapeutic target, which also expands the mechanisms and clinical roles of circRNAs in RCC.

## Results

### Decreased expression of hsa_circ_0060927 (circCYP24A1) is observed in RCC and correlates with an unfavorable prognosis

**Fig. 1:**
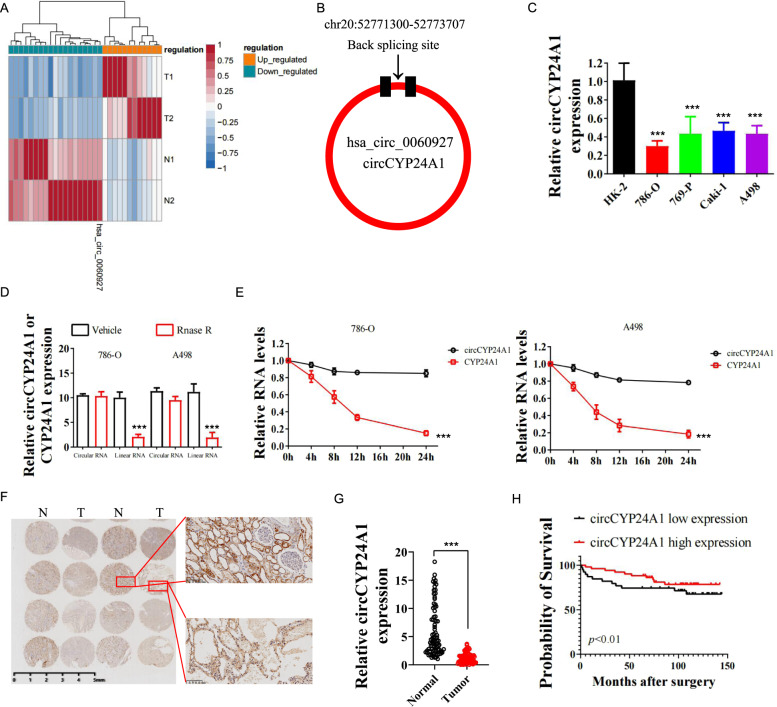
The circRNA circCYP24A1 is downregulated in RCC and associated with a favorable prognosis for patients with RCC. **A** The differential expression analysis showed that circRNAs were differentially expressed in RCC tumor and normal tissues (*P* < 0.05 and |log2FC| > 1.5). Notably, hsa_circ_0060927 was identified as the most highly differentially expressed circRNA. **B** The location and illustration of hsa_circ_0060927 (circCYP24A1). **C** Relative expression of circCYP24A1 in the RCC cell lines 786-O, 769-P, Caki-1, and A498 was evaluated and compared with the normal kidney proximal tubular cell line HK-2 using qRT-PCR. **D** qRT-PCR was performed to measure the relative expression of CYP24A1 and circCYP24A1 in 786-O and A498 cell lines treated with RNase R or vehicle control. **E** Relative expression of circular and linear CYP24A1 in 786-O and A498 cells incubated with actinomycin. **F**, **G** In situ hybridization was performed to assess the expression of circCYP24A1 in tumor and normal tissues from patients with RCC. **H** Kaplan–Meier analysis was conducted to measure the survival rate of patients with RCC presenting high or low circCYP24A1 expression. Data are presented as means ± SD. ***p < 0.001.

High-throughput sequencing was performed to discover the differential expression of circRNAs in 2 pairs of fresh RCC and peritumor normal tissues. Twelve circRNAs were upregulated, while 19 circRNAs were downregulated in RCC tissues compared with normal tissues according to our analysis of the sequencing data (Fig. 1A). Among the 19 decreased circRNAs in RCC, hsa_circ_0060927 (circCYP24A1) was one of the most substantially downregulated circRNAs and was derived from the CYP24A1 gene located on chr20:52771300–52773707 (Fig. 1B). Moreover, the RCC tumor cell lines 786-O, 769-P, Caki-1, and A498 expressed circCYP24A1 at lower levels than the normal cell line HK-2 (Fig. 1C), consistent with our findings from clinical tumor samples. circCYP24A1 showed more resistance to RNase R than linear CYP24A1 in 786-O and A498 cell lines (Fig. 1D). In addition, the circRNA form of CYP24A1 showed relatively higher stability than its linear form after actinomycin-D treatment (Fig. 1E). CircCYP24A1 expression was then assessed in the RCC TMA of 82 patients who underwent nephrectomy (Fig. 1F). We found that the RCC tumor tissue expressed a decreased level of circCYP24A1 compared with the peritumor normal tissue (Fig. 1G). K–M analysis revealed that patients with RCC presenting low circCYP24A1 expression experienced shorter overall survival (Fig. 1H) and a higher tumor burden and tumor grades (Table 1). Overall, the lack of circCYP24A1 in RCC was associated with worse clinical outcomes, indicating its crucial role in tumorigenesis.

**Table 1:** Relationship between the expression levels of circCYP24A1 and clinicopathological features in RCC.

Characteristics	No. (%)	circCYP24A1 expression		
		High (%)	Low (%)	*P*-value
*Gender*				
Male	46 (56.1)	20 (43.5)	26 (56.5)	0.557
Female	36 (43.9)	18 (50.0)	18 (50.0)	
*Age*				
<60	35 (42.7)	14 (40.0)	21 (60.0)	0.320
≥60	47 (57.3)	24 (51.1)	23 (48.9)	
*Tumor size*				
<3 cm	33 (40.2)	20 (60.6)	13 (39.4)	0.034
≥3 cm	49 (50.8)	18 (36.7)	31 (63.3)	
*TNM stage*				
I	72 (87.8)	37 (51.4)	35 (48.6)	0.014
II–IV	10 (12.2)	1 (10.0)	9 (90.0)	
Total	82	38	44	

### Overexpression of circCYP24A1 inhibits the proliferation, invasion, and migration of RCC cells

**Fig. 2:**
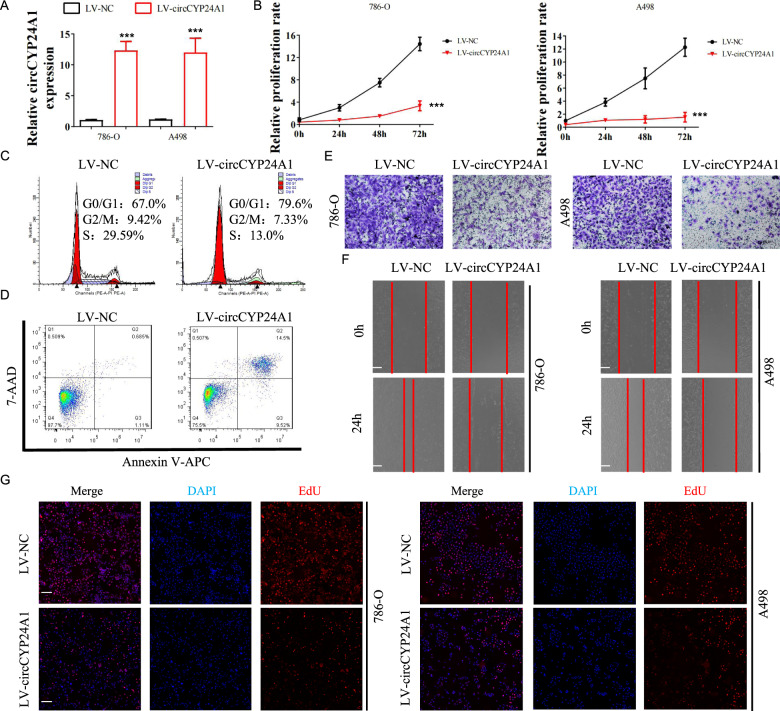
Overexpression of circCYP24A1 inhibits the tumorigenesis of RCC cells in vitro. **A** Relative expression of circCYP24A1 was evaluated using qRT-PCR in 786-O and A498 cell lines transfected with LV-NC or LV-circCYP24A1. **B** Relative proliferation rates were evaluated in 786-O and A498 cell lines transfected with LV-NC or LV-circCYP24A1. The cell cycle distribution (**C**) and apoptosis rate (**D**) were evaluated in 786-O cell lines transfected with LV-NC or LV-circCYP24A1 using flow cytometry. Transwell assays (**E**) and wound-healing assays (**F**) were performed in 786-O and A498 cell lines transfected with LV-NC or LV-circCYP24A1. The bar corresponds to 300 μm in the wound-healing assay. **G** An EdU assay was performed to measure the proliferation of 786-O and A498 cell lines transfected with LV-NC or LV-circCYP24A1. The bar corresponds to 100 μm. Data are presented as means ± SD. ***p < 0.001.

Next, we evaluated whether circCYP24A1 regulated the tumorigenesis of RCC. A lentivirus vector for circCYP24A1 overexpression (LV-circCYP24A1) was constructed. We confirmed the elevated expression of circCYP24A1 in 786-O and A498 cell lines transfected with LV-circCYP24A1 (Fig. 2A). These two cell lines with circCYP24A1 overexpression showed significantly reduced proliferation in CCK-8 assays (Fig. 2B). The cell cycle analysis revealed an increased proportion of RCC cells transfected with LV-circCYP24A1 in S phase but a decreased proportion in G0/G1 phase (Fig. 2C, Supplementary Fig. 1A). Moreover, more apoptotic RCC cells were detected after circCYP24A1 overexpression, indicating that circCYP24A1 promoted the apoptosis of RCC (Fig. 2D, Supplementary Fig. 1B). Transwell assays (Fig. 2E, Supplementary Fig. 1C) and wound-healing assays (Fig. 2F, Supplementary Fig. 1D) showed that RCC cells overexpressing circCYP24A1 exhibited relatively low invasion and migration levels. Additionally, tumor cells transfected with LV-circCYP24A1 showed lower proliferation rates than the control cells (Fig. 2G, Supplementary Fig. 1E). Based on these results, circCYP24A1 overexpression suppressed the proliferation, invasion, and migration of RCC cells.

### CircCYP24A1 functions as a sponge for miR-421

**Fig. 3:**
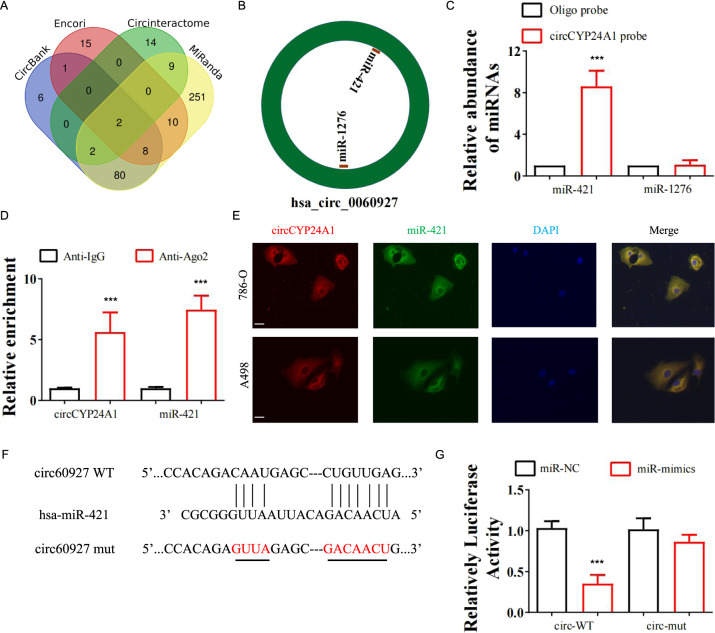
CircCYP24A1 functions as a sponge for miR-421 in RCC cells. **A** The miRNAs that potentially interact with circCYP24A1 were screened using four databases: CircBank, Encori, CircInteractome, and MiRanda. **B** Screening of the four databases identified miR-421 and miR-1276 as two possible targets of circCYP24A1. **C** Relative expression of miR-421 and miR-1276 was measured using qRT-PCR after RNA pulldown with the circCYP24A1 probe or the control probe in 786-O cells. **D** Ago2 RIP was performed to detect the precipitation of circCYP24A1 and miR-421. **E** CircCYP24A1 and miR-421 were detected by FISH in 786-O and A498 cells. Nuclei were stained with DAPI. The bar corresponds to 20 μm. **F** Mutant and wild-type circCYP24A1 sequences were constructed for the dual-luciferase reporter assay. **G** Relative luciferase activity was measured in 786-O cells transfected with miR-421 mimics or normal control along with mutant or wild-type circCYP24A1. Data are presented as means ± SD. ***p < 0.001.

CircRNAs are known to influence the expression of downstream mRNAs by functioning as miRNA sponges [14]. Thus, we explored the targets of circCYP24A1 to further elucidate its effect on tumor progression by examining four online prediction databases, including circBank, Encori, CircInteractome, and MiRanda (Fig. 3A). Two miRNAs (miR-1276 and miR-421) were selected after screening (Fig. 3A, B). A strong interaction was identified between circCYP24A1 and miR-421, but not miR-1276, using RNA-pulldown assays (Fig. 3C). Ago2 RNA immunoprecipitation validated that circCYP24A1 and miR-421 bound to Ago2 to form an Ago2-regulated complex (Fig. 3D). The colocalization of circCYP24A1 and miR-421 was also observed in the cytoplasm (Fig. 3E), suggesting that miR-421 was the binding target of circCYP24A1. A dual-luciferase reporter assay was performed and showed that transfection of miR-421 mimics decreased the luciferase activity of wild-type circCYP24A1 (Fig. 3F), while miR-421 mimics failed to reduce the luciferase activity of mutant circCYP24-A1 (Fig. 3G). Thus, we suggested that circCYP24 A1 served as an miR-421 sponge in RCC cells.

### CMTM4 is the target of miR-421 in RCC and correlates with favorable clinical outcomes

**Fig. 4:**
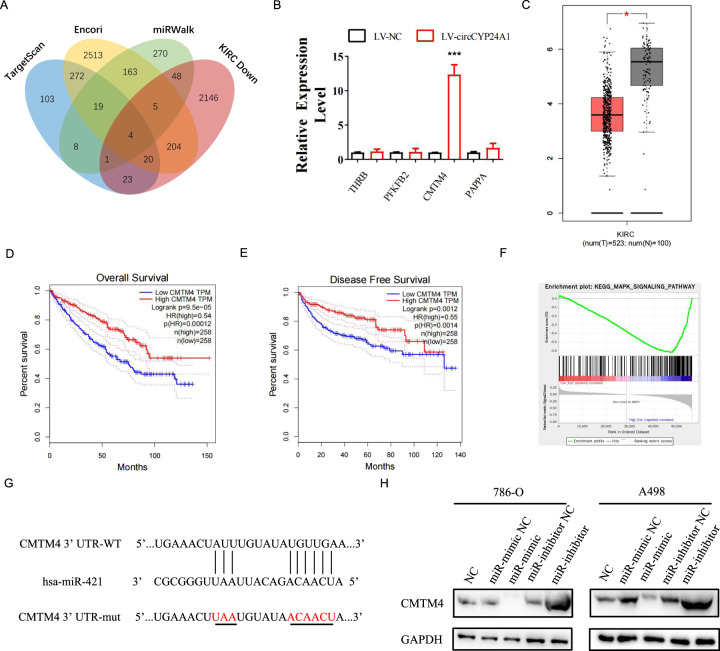
CMTM4 is the target of miR-421 in RCC. **A** The possible targets of miR-421 were predicted by four databases, including TargetScan, Encori, miRWalk, and KIRC Down. KIRC Down represents the downregulated genes in the differential gene expression analysis of TCGA-KIRC database. **B** Relative expression of THRB, PFKFB2, CMTM4, and PAPPA was measured using qRT-PCR in 786-O cells transfected with LV-NC or LV-circCYP24A1. **C** CMTM4 TPM expression in the tumor tissues compared with the normal tissues in TCGA-KIRC database. Overall survival (**D**) and disease-free survival (**E**) were measured using the Kaplan–Meier analysis. **F** Gene-set enrichment analysis of the MAPK signaling pathway in patients from TCGA-KIRC with high/low CMTM4 expression. **G** The predicted binding site for circCYP24A1 and miR-421 is illustrated. Mutant and wild-type CMTM4 constructs for dual-luciferase reporter assays were generated. **H** CMTM4 expression was detected using Western blotting in 786-O and A498 cells transfected with miR-421 mimic, miR-421 inhibitor, or its normal control. Data are presented as means ± SD. ***p < 0.001.

Four databases, TargetScan, Encori, miRWalk, and TCGA-KIRC, were analyzed for cross-matching to identify the potential targets of miR-421 in RCC cells (Fig. 4A). Four potential targets (THRB, PFKB2, CMTM4, and PAPPA) were identified, but only CMTM4 expression increased with the overexpression of circCYP24A1 (Fig. 4B), indicating that CMTM4 was the target of miR-421 in RCC cells. CMTM4 expression decreased in RCC tumors compared with normal tissues in TCGA-KIRC database (Fig. 4C). Moreover, patients with RCC presenting high CMTM4 expression exhibited longer overall survival and disease-free survival than patients with low CMTM4 expression (Fig. 4D, E). Gene-set enrichment analysis showed that CMTM4 expression was negatively associated with the MAPK signaling pathway, suggesting that CMTM4 may regulate this pathway to suppress tumor progression. Dual-luciferase reporter vectors for wild-type and mutant CMTM4 were constructed to further confirm the effect of miR-421 on CMTM4 expression (Fig. 4G, Supplementary Fig. 1F). After the transfection of miR-421 mimics and miR-421 inhibitor, CMTM4 expression decreased in RCC cells from the miR-mimic transfection group and increased in the miR-inhibitor-treated group (Fig. 4H), indicating that CMTM4 served as the direct target of miR-421.

### Overexpression of miR-421 and silencing of CMTM4 reverse the suppression of the malignant phenotype induced by circCYP24A1 overexpression

**Fig. 5:**
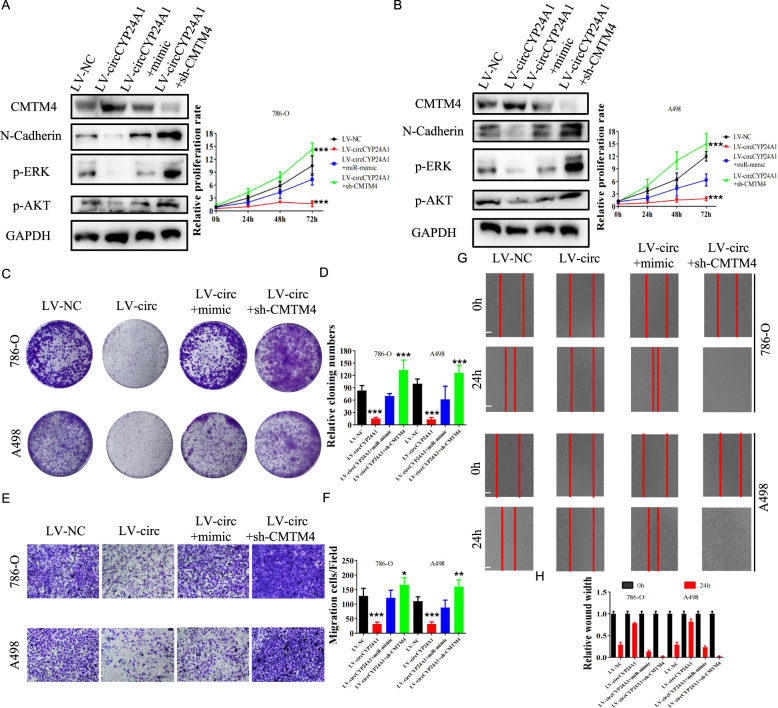
Increases in proliferation, invasion, and migration mediated by CircCYP24A1 overexpression were abrogated in RCC cells transfected with the miR-421 mimic or sh-CMTM4. The levels of N-cadherin (EMT signaling pathway), p-ERK (MEK/ERK pathway), and p-AKT (AKT/mTOR pathway) were evaluated using Western blotting, and the relative proliferation rate of cells was measured in 786-O (**A**) and A498 (**B**) cells transfected with LV-NC, LV-circCYP24A1, LV-cirCYP24A1+miR-421 mimic and LV-circCYP24A1+sh-CMTM4. Colony-formation assay (**C**, **D**), Transwell invasion assay (**E**, **F**) and wound-healing assay (**G**, **H**) were performed and quantitatively analyzed in 786-O and A498 cells transfected with LV-NC, LV-circCYP24A1, LV-cirCYP24A1 plus miR-421 mimic, and LV-circCYP24A1 plus sh-CMTM4. Data are presented as means ± SD. ***p < 0.001. The bar corresponds to 300 μm in the wound-healing assay.

The association between the circCYP24A1/miR-421/CMTM4 pathway and tumor progression was further explored. Levels of proteins involved in EMT signaling (N-cadherin), MEK–ERK signaling (p-ERK), and AKT/mTOR signaling (p-AKT) were detected in 786-O and A498 cells transfected with the circCYP24A1-overexpression construct, miR-421 mimics, or sh-CMTM4 using Western blotting. Notably, circCYP24A1 overexpression significantly abrogated RCC malignant phenotype-related pathways and proliferation, while miR-421 overexpression and CMTM4 silencing rescued these changes (Fig. 5A, B). Colony-formation assays (Fig. 5C and D) and Transwell assays (Fig. 5E and F) further showed that miR-421 overexpression and CMTM4 silencing promoted tumor proliferation and invasion after circCYP24A1 overexpression. Consistent with the aforementioned results, wound-healing assays also revealed increased migration of RCC cells cotransfected with LV-circCYP24A1 and miR-421 mimics or sh-CMTM4 (Fig. 5G, H). In conclusion, overexpression of miR-421 and silencing of CMTM4 reversed tumor suppression induced by circCYP24A1 expression.

### MiR-421 promotes RCC tumor progression by inhibiting CMTM4

**Fig. 6:**
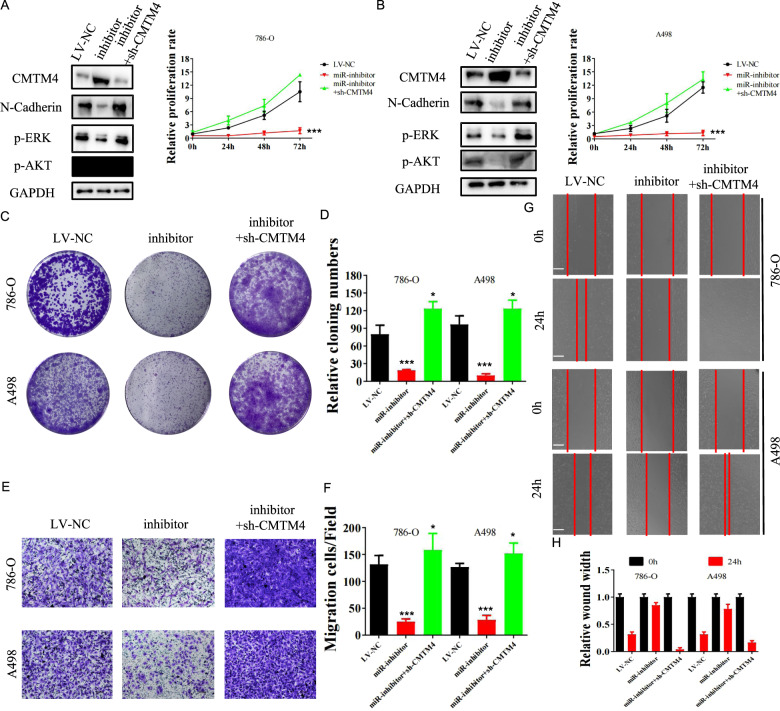
Inhibition of miR-421 suppresses the proliferation, invasion, and migration of RCC cells in vitro, which were reversed by silencing CMTM4. The levels of N-cadherin (EMT signaling pathway), p-ERK (MEK/ERK pathway), and p-AKT (AKT/mTOR pathway) were evaluated using Western blotting, and the relative proliferation rate of 786-O (**A**) and A498 (**B**) cells transfected with LV-NC, miR-421 inhibitor, and miR-421 inhibitor+sh-CMTM4 was measured. Colony-formation assays (**C**, **D**), transwell assays (**E**, **F**), and wound-healing assays (**G**, **H**) were performed and quantitatively analyzed in 786-O cells transfected with LV-NC, miR-421 inhibitor, and miR-421 inhibitor plus sh-CMTM4. Data are presented as means ± SD. ***p < 0.001. The bar corresponds to 300 μm in the wound-healing assay.

The oncogenic function of miR-421 through CMTM4 inhibition was further explored. 786-O and A498 cell lines were treated with miR-412 inhibitor or inhibitor plus sh-CMTM4. EMT signaling, MEK/ERK signaling, AKT/mTOR signaling, and proliferation were constrained in cells transfected with the miR-412 inhibitor but activated in cells transfected with the inhibitor plus sh-CMTM4 (Fig. 6A, B). Moreover, colony-formation assays (Fig. 6C, D) and Transwell assays (Fig. 6E, F) indicated the tumor-suppression capacity of the miR-421 inhibitor, while this effect was diminished with the addition of sh-CMTM4. The migration of 786-O and A498 cells was also suppressed by the miR-421 inhibitor, while the effect was abolished by the addition of sh-CMTM4 (Fig. 6G, H).

### Overexpression of circCYP24A1 leads to tumor suppression in vivo, but the changes are reversed after miR-421 overexpression or CMTM4 silencing

**Fig. 7:**
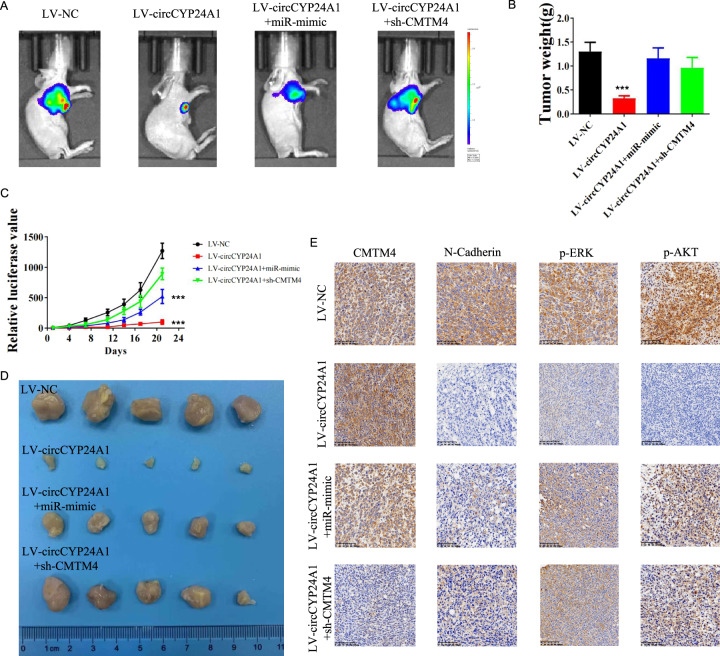
Overexpression of circCYP24A1 suppresses RCC progression in vivo. Luciferase-expressing 786-O cells transfected with LV-NC, LV-circCYP24A1, LV-cirCYP24A1+miR-421 mimic, and LV-circCYP24A1+sh-CMTM4 were used to establish subcutaneous xenograft models (*N* = 5). **A** Tumor growth was monitored using in vivo bioluminescence imaging. **B** Weights of subcutaneous 786-O tumors were evaluated 21 days after implantation. **C** Relative luciferase values of subcutaneous 786-O tumors were assessed. **D** Subcutaneous 786-O tumors are shown. **E** Immunohistochemical staining for CMTM4, N-cadherin, p-ERK, and p-AKT was performed in the subcutaneous tumors from the 4 groups.

We further validated the role of the circCYP24A1/miR-421/CMTM4 axis in RCC in vivo. 786-O-luc cells transfected with LV-NC, LV-circCYP24A1, LV-circCYP24A1 plus miR-421 mimic, and LV-circCYP24A1 plus sh-CMTM4 were subcutaneously injected into nude mice and tumor growth was evaluated (Fig. 7A). Overexpression of circCYP24A1 significantly suppressed tumor growth in vivo and was reversed by the addition of miR-421 inhibitor or sh-CMTM4 (Fig. 7B–D). Consistent with our previous findings, N-cadherin (EMT marker), p-ERK (MEK/ERK signaling marker), and p-AKT (AKT/mTOR signaling marker) levels were reduced in tumors with circCYP24A1 overexpression and restored by the addition of miR-421 inhibitor or sh-CMTM4 (Fig. 7E). Therefore, circCYP24A1 overexpression inhibited tumor progression in vivo through the miR-421/CMTM4 pathway.

### circCYP24A1 overexpression in RCC cells leads to reduced lung metastasis

**Fig. 8:**
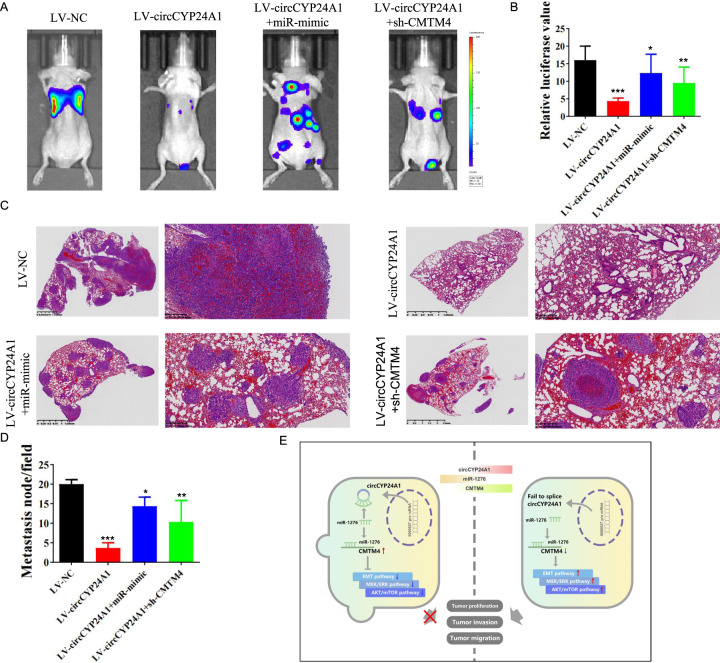
Overexpression of circCYP24A1 restrains RCC metastasis. **A** Bioluminescence imaging showed the RCC metastasis sites in the lungs of mice implanted with 786-O cells transfected with LV-NC, LV-circCYP24A1, LV-cirCYP24A1+miR-421 mimic, and LV-circCYP24A1+sh-CMTM4 (*N* = 5). **B** Relative luciferase values of 786-O metastatic lesions in the lung were assessed 21 days after intravenous injection. **C** Representative images of hematoxylin and eosin staining of lung metastasis sites are illustrated. **D** Metastatic nodules of RCC were analyzed in the 4 groups. **E** Schematic diagram of the function of circCYP24A1/miR-421/CMTM4 in RCC.

As RCC shows a relatively high metastasis rate, we further explored whether circCYP24A1 overexpression correlates with the metastatic ability of RCC cells. 786-O-luc cells transfected with LV-NC, LV-circCYP24A1, LV-circCYP24A1 plus miR-421 mimic, and LV-circCYP24A1 plus sh-CMTM4 were injected into the tail vein of nude mice to explore the lung metastasis ability of RCC cells. Bioluminescence imaging showed a decreased number of metastatic lesions in mice with circCYP24A1 overexpression, but an increased number in mice with miR-421 overexpression or CMTM4 knockdown (Fig. 8A, B). The visible metastatic lesions in the lungs were assessed and showed a reduction in the circCYP24A1-overexpression group. The metastatic lesions in mice treated with LV-circCYP24A1 plus miR-421 mimic and LV-circCYP24A1 plus sh-CMTM4 were also decreased compared with those of the control mice (Fig. 8C, D). Thus, we suggested that circCYP24A1 overexpression suppressed tumor metastasis in vivo by regulating the circCYP24A1/miR-421/CMTM4 network in RCC (Fig. 8E).

## Discussion

Local tumor progression and distant metastasis of RCC are the leading causes of mortality in patients with RCC, which encouraged us to discover the molecular landscapes of the key regulatory mechanism involved in the invasiveness and metastatic behaviors of RCC. Several circRNAs have been identified with the development of sequencing strategies and are characterized by stable closed-ring structures [15] and location-related widespread functions in human tissues [16]. Notably, circRNAs function as miRNA sponges [4, 17] and interact with proteins [18–20] in the cytoplasm while possessing the ability to be translated [5]. The miRNA sponge function of ceRNA-regulatory networks has been extensively analyzed and is a widely recognized regulatory mechanism.

Thus, in our study, we analyzed circRNA expression in 2 pairs of RCC tissues and matched normal tissues and found that circCYP24A1 was downregulated in RCC and associated with a better prognosis. Furthermore, circCYP24A1 modulated RCC proliferation and invasion in vitro and in vivo. In cancer, miR-421 has been identified as a tumor promoter in osteosarcoma [10] and lung cancer [11], while its role in RCC has not been studied. Here, we confirmed that miR-421 overexpression promoted the proliferation and invasion of RCC cells. RIP, RNA pulldown, and dual-luciferase assays also supported the prediction that miR-421 is a target of circCYP24A1. We first documented the antitumor activity of circCYP24A1 in RCC and identified miR-421 as its downstream target through a combination of bioinformatics and experimental analyses.

The human chemokine-like factor (CKLF)-like MARVEL transmembrane-domain-containing family (CMTM) plays an important role in human cancer, and CMTM4 is the most conserved member in the CMTM family. Several studies have identified CMTM4 as a tumor suppressor in various types of malignant lesions [12, 13], while the regulatory role of CMTM4 in RCC has not been studied. Here, higher CMTM4 expression inhibited EMT in RCC and altered the cellular phenotype. Additionally, we identified that CMTM4 correlated with the MAPK pathway by performing a GSEA of RCC. We further confirmed that CMTM4 overexpression also inhibited the MEK/ERK and AKT/mTOR pathways, while miR-421 and circCYP24A1 exerted the opposite effect. Since the MEK/ERK and AKT/mTOR pathways regulate the key metabolic process in carcinogenesis, approaches targeting the circCYP24A1/miR-421/CMTM4 pathway may become a promising treatment strategy in RCC. In vivo animal experiments also proved that circCYP24A1 overexpression led to impaired proliferation and metastasis of RCC by reducing the activity of the MEK/ERK and AKT/mTOR pathways. Generally, we confirmed CMTM4 as a tumor suppressor in RCC and a downstream target of circCYP24A1/miR-421.

In conclusion, the main contribution and novel finding of our work is the identification of circCYP24A1 as a biomarker and regulator of the malignant phenotype of RCC, which might benefit therapeutic approaches using exosome-based treatments. However, the mechanism of circCYP24A1 in RCC should be discovered in future in-depth research. Overall, we identified circCYP24A1/miR-421/CMTM4 as a novel regulatory pathway in RCC and circCYP24A1 as a potential prognosis-related biomarker in RCC.

## Materials and methods

### Patient samples and circRNA sequencing

After receiving approval from the Ethics Committee, renal cancer and paired normal tissue samples were obtained and snap-frozen in liquid nitrogen. Total RNA was extracted, treated with RNase R, and then subjected to high-throughput sequencing using standard procedures described in our previous study [21]. CircRNAs expressed in the tissues were detected, and differential gene expression analysis was performed. The differentially expressed circRNAs were defined based on *P* < 0.05 and |lgo2FC| > 1.5. The project was approved by the Board and Ethics Committee of Huashan Hospital, Fudan University (Approval number KY2011-009). Written informed consent was acquired from each participant.

### Quantitative reverse-transcription PCR (qRT-PCR)

Total RNA was first isolated from tissues or cells with TRIzol reagent (Thermo Fisher Scientific, Invitrogen) according to the manufacturer’s instructions. SuperScript II Reverse Transcriptase (Thermo Fisher Scientific, Invitrogen) was applied to reverse-transcribe RNA into cDNAs. qRT-PCR was then performed using an ABI PRISM 7900HT Sequence Detection System (Thermo Fisher Scientific, Invitrogen). GAPDH was selected as the reference gene for circRNAs and mRNAs. U6 was selected as the internal control gene for miRNAs.

### Cell transfection and vector construction

All cells were obtained from ATCC and authenticated through STR profiling and tested negative for mycoplasma. Lipofectamine 2000 (Invitrogen, USA) was used to transfect shRNAs, siRNAs, and miRNA mimics and inhibitors (GenePharma, Shanghai, China) into cells. The sequence of circCYP24A1 was cloned into a plenti-ciR-GFP-T2A vector to construct the overexpression vector.

### RNA in situ hybridization (ISH) and immunohistochemistry (IHC)

CircCYP24A1 expression in ccRCC was determined by hybridizing biotin-labeled probes with a tissue microarray (TMA) as described previously [22]. Tumor tissue samples were embedded in paraffin and stained with protein markers as described previously [23].

### RNA fluorescence in situ hybridization (FISH)

Specific probes for circCYP24A1 and miR-421 were prepared (Geneseed Biotech, Guangzhou, China). FISH was performed as described in previous studies [24].

### RIP assay

The RIP assay was performed with the Magnetic RIP RNA-binding protein immunoprecipitation kit (Millipore) as previously described [23].

### RNA pulldown

Probe-coated beads were constructed by incubating biotin-coupled circCYP24A1 or oligo probes with streptavidin-conjugated magnetic beads (Life Technologies, USA) for 3 h at 25 °C, which were used for RNA-pulldown analysis described in a previous study [23].

### Western blotting analysis

Protein was extracted from cells that had been lysed in ice-cold RIPA lysis buffer, separated on SDS-PAGE gels, and incubated with antibodies against specific proteins using the methods described in our previous study. CMTM-4 (Abcam, ab254657), N-cadherin (CST, 13116), p-AKT (CST, 4060), p-ERK (CST, 4370), and GAPDH (Proteintech, 60004-1-lg) antibodies were selected for the WB assay.

### Cell counting Kit-8 assay

A Cell Counting Kit-8 assay (CCK-8, Sigma-Aldrich) was applied to assess cell proliferation. Cells were seeded in 96-well plates at a density of 1500 cells per well. Cell viability was evaluated at 0, 24, 48, and 72 h after cell seeding.

### Colony-formation assay

Cells were plated in 6-well plates at a density of 600 cells per well. The colonies were then fixed and stained after 8 days of culture in DMEM plus 10% FBS.

### Apoptosis detection

An Annexin V-APC/7-AAD apoptosis kit (MultiSciences, AP105-100) was applied to detect apoptotic cells according to the manufacturer’s procedure. Samples were separated using a BD Accuri C6 flow cytometer (BD Biosciences), and the data were subsequently analyzed using FlowJo software. Annexin-V+ 7-AAD- cells were defined as viable apoptotic cells. Annexin-V+ 7-AAD+ cells were defined as nonviable nonapoptotic cells.

### Transwell assay

Cell invasion was assessed using Transwell chambers (Costar) in 24-well plates according to the manufacturer’s procedure. A total of 60,000 cells in 600 µl of serum-free medium were seeded in the upper chambers, while medium containing 10% FBS was added to the lower chambers. Invading cells were stained with 1% crystal violet and evaluated after a 24 h incubation at 37 °C.

### Wound-healing assay

A total of 30,000 cells were seeded in each well, and a straight scratch was created with a pipette tip. Cell proliferation was inhibited by culturing cells with DMEM plus 5 µg/ml mitomycin C. The wounds were evaluated 24 h after scratching.

### Luciferase-reporter assay

Wild-type and mutant fragments of the circCYP24A1 cDNA and CMTM4 3′-UTR were amplified by PCR and cloned into psiCHECK-2 (Promega, Madison, WI, USA) as previously described [21].

### Animal study

Animal experiments were performed according to the guidelines of the Ethics Committee of Fudan University (Approval number 2021JS-173). A total of 1 × 10^7^ LV-NC or LV-circCYP24A1 786-O cells were subcutaneously injected into the flanks of 4-week-old nude mice. For each treatment group, the sample size was determined to be six. All animals were ranked and selected for each group according to their weight. Tumor growth was monitored every 5 days. After 3 weeks, the mice were sacrificed and the tumors were excised and weighed.

For analysis of metastasis, LV-NC or LV-circCYP24A1 786-O-luc cells (2 × 10^5^) were injected through the tail vein into six randomly selected nude mice. After 30 days, the metastasis of 786-O cells was identified by performing a bioluminescence imaging analysis.

### Bioinformatics analysis

Seven databases, including CircBank, Encori, CircInteractome, MiRanda, TargetScan, miRWalk, and TCGA, were applied in the study to assess circRNA/miRNA and miRNA/mRNA interactions. Patients in TCGA-KIRC were divided into high/low groups based on the median expression of CMTM4 TPM. Kaplan–Meier analysis was performed to compare the clinical outcomes of the 2 groups. GSEA was conducted to explore the related pathways in the 2 groups. The sample size for analysis was three patients per treatment group.

## Supplementary information

Supplementary figure 1

Language Certification

Reproducibility checklist

Supplementary primer

## Data Availability

The original data of the study can be found in the article/Supplementary Material, further inquiries can be directed to the corresponding authors.
