# Supplementary material for: CircCYP24A1 hampered malignant phenotype of renal cancer carcinoma through modulating CMTM-4 expression via sponging miR-421
Source: Cell Death Dis. 2022 Feb 26;13(2):190. doi: 10.1038/s41419-022-04623-0 (PMC8882186; doi:10.1038/s41419-022-04623-0)
Supplement: Supplementary file 1 — Supplementary figure 1 [file 41419_2022_4623_MOESM1_ESM.docx]

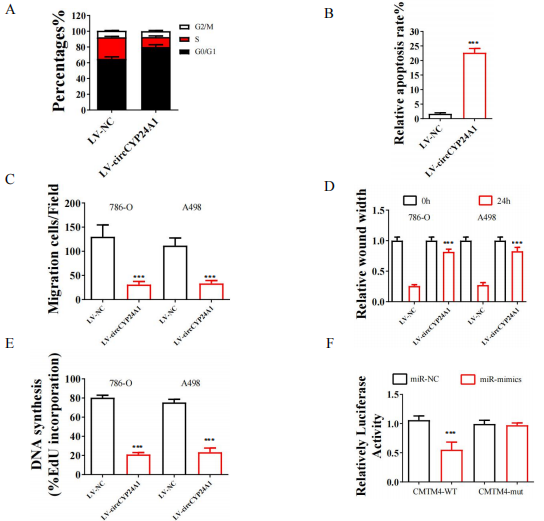


**Supplementary Figure 1**

(A) The cell cycle distribution was evaluated in 786-O cell lines transfected with LV-NC or LV-circCYP24A1 using flow cytometry. (B) The apoptosis rate was evaluated in 786-O cell lines transfected with LV-NC or LV-circCYP24A1 using flow cytometry. (C) Transwell assays were performed in 786-O and A498 cell lines transfected with LV-NC or LV-circCYP24A1. (D) Wound healing assays were performed in 786-O and A498 cell lines transfected with LV-NC or LV-circCYP24A1. (E) An EdU assay was conducted to measure the proliferation of 786-O and A498 cells transfected with LV-NC or LV-circCYP24A1. (F) Relative luciferase activity in HEK293T cells after transfection with miRNA mimic/negative control or with CMTM4 WT/Mut. Data are presented as means±SD. ***, p<0.001.
