# Supplementary material for: CircCYP24A1 hampered malignant phenotype of renal cancer carcinoma through modulating CMTM-4 expression via sponging miR-421
Source: Cell Death Dis. 2022 Feb 26;13(2):190. doi: 10.1038/s41419-022-04623-0 (PMC8882186; doi:10.1038/s41419-022-04623-0)
Supplement: Supplementary file 4 — Supplementary primer [file 41419_2022_4623_MOESM4_ESM.doc]

**RT-qPCR primers:**

GAPDH Forward 5-CAAGGCTGAGAACGGGAAG-3

GAPDH Reverse 5-TGAAGACGCCAGTGGACTC-3

CYP24A1 Forward 5-GGTGGCGAGACTCAGAACG-3

CYP24A1 Reverse 5-GTCGTGCTGTTTCTTGAGACC-3

circCYP24A1 Forward 5-GTTGTCCAGCTTCATCACTT-3

circCYP24A1 Reverse 5-GATTGTCCGCAAATACGA-3

THRB Forward 5-TGGGACAAACCGAAGCACTG-3

THRB Reverse 5-TGGCTCTTCCTATGTAGGCAG-3

CMTM4 Forward 5-CAAGGTCGCCCAAGTGATCTT-3

CMTM4 Reverse 5-GTGCAGGTTGAGACTGAACATA-3

PFKFB2 Forward 5-TGGGCCTCCTACATGACCAA-3

PFKFB2 Reverse 5-CAGTTGAGGTAGCGTGTTAGTTT-3

PAPPA Forward 5-ACAAAGACCCACGCTACTTTTT-3

PAPPA Reverse 5-CATGAACTGCCCATCATAGGTG-3

miR-421 5-ACACTCCAGCTGGGATCAACAGACATTAATT-3

miR-1276 5-ACACTCCAGCTGGGTAAAGACCCCTGTG-3

miRNA reverse primer 5-TGGTGTCGTGGAGTCG-3

U6 Forward 5-CTCGCTTCGGCAGCACA-3

U6 Reverse 5-AACGCTTCACGAATTTGCGT-3

**Probes:**

circCYP24A1-Cy3: 5-CAGTCTTCCCCTTCCCTGAGGCGTATTATC-3

circCYP24A1-Bio: 5-CAGTCTTCCCCTTCCCTGAGGCGTATTATC-3

miR-421-Dig: 5-TAGTTGTCTGTAATTAACCCGCG-3
